# Differential expression of P2X7 receptor and IL-1β in nociceptive and neuropathic pain

**DOI:** 10.1186/s12974-016-0565-z

**Published:** 2016-05-04

**Authors:** Benjamin Luchting, Jens Heyn, Tobias Woehrle, Banafscheh Rachinger-Adam, Simone Kreth, Ludwig C Hinske, Shahnaz C Azad

**Affiliations:** Department of Anesthesiology and Pain Medicine, LMU-Munich, Marchioninistrasse 15, 81377 Munich, Germany

**Keywords:** Neuropathic pain, Chronic low back pain, T cells, TH17, Treg, Il-1β, Neuroinflammation

## Abstract

**Background:**

Despite substantial progress, pathogenesis and therapy of chronic pain are still the focus of many investigations. The ATP-gated P2X7 receptor (P2X7R) has previously been shown to play a central role in animal models of nociceptive inflammatory and neuropathic pain. Recently, we found that the adaptive immune system is involved in the pathophysiology of chronic nociceptive and neuropathic pain in humans. So far, data regarding P2X7R expression patterns on cells of the adaptive immune system of pain patients are scarce. We therefore analyzed the P2X7R expression on peripheral blood lymphocytes and monocytes, as well as serum levels of IL-1β in patients suffering from chronic nociceptive and neuropathic pain in comparison to healthy volunteers in order to identify individuals who might benefit from a P2X7R modulating therapy.

**Methods:**

P2X7R messenger RNA (mRNA) and protein expression were determined in patients with either chronic nociceptive low back pain (CLBP) or neuropathic pain (NeP), and in healthy volunteers by quantitative real-time PCR (qPCR) and by fluorescence-assisted cell-sorting (FACS), respectively. IL-1β serum levels were measured with a multiplex cytokine assay.

**Results:**

Compared to healthy volunteers, P2X7R mRNA (1.6-fold, *p* = 0.038) and protein levels were significantly increased on monocytes (NeP: 24.6 ± 6.2, healthy volunteers: 17.0 ± 5.4; *p* = 0.002) and lymphocytes (NeP: 21.8 ± 6.5, healthy volunteers: 15.6 ± 5.2; *p* = 0.009) of patients with NeP, but not in patients with CLBP. Similarly, IL-1β serum concentrations were significantly elevated only in NeP patients (1.4-fold, *p* = 0.04).

**Conclusions:**

A significant upregulation of P2X7R and increased IL-1β release seems to be a particular phenomenon in patients with NeP. P2X7R inhibitors may therefore represent a potential option for the treatment of this frequently intractable type of pain.

German Clinical Trial Register (DRKS): Registration Trial DRKS00005954.

## Background

Purinergic P2X receptors (P2XRs) are ATP-gated cation channels, divided into seven subtypes (P2XR1-P2XR7). They are predominantly expressed on cells of the hematopoietic lineage including macrophages, microglia, and lymphocyte subtypes [1].

The subtype receptor P2X7R has been found to play a major role in central nervous system (CNS) processes, including neurodegeneration, traumatic brain injuries, psychiatric disorders, and pain. The exceptional role of P2X7R in pain syndromes has been shown in various animal models of nociceptive inflammatory and neuropathic pain [2–5]. In humans, P2X7R messenger RNA (mRNA) expression was shown to be increased in leukocytes of patients with chronic fatigue syndrome [6]. Results of experimental P2X7R inhibition in a rat model of inflammatory arthritis, induced by injection of Freund’s complete adjuvant into one hind paw, were promising [7]. Furthermore, P2X7R antagonists have been tested in clinical trials in patients with rheumatoid arthritis [8, 9]. P2X7R activation leads to a rapid increase in intracellular calcium concentrations and triggers the release of the pro-inflammatory and pro-nociceptive cytokine IL-1β [10]. IL-1β is known to be a key mediator in neurodegeneration, chronic inflammation, and chronic pain by affecting neuronal cell death [1, 10]. Pharmacological inhibition of IL-1β attenuates hyperalgesia induced by spinal cord inflammation in rats [11]. Moreover, higher serum levels of IL-1β have been implicated in the pathogenesis of depression [12, 13]. Accordingly, P2X7R inhibitors displayed an antidepressant activity in mice [14]. P2X7R has been found to be readily expressed on the cell surface of both microglia and immune cells, suggesting a link between the CNS and the immune system [1, 15]. Since the adaptive immune system is critically involved in the pathophysiology of chronic pain [16], we systematically investigated the expression of P2X7R on lymphocytes and monocytes of patients suffering from either chronic low back pain (CLBP) or chronic neuropathic pain (NeP) in comparison to healthy volunteers. We analyzed P2X7R protein by flow cytometry (FACS) and determined P2RX7 mRNA expression by real-time PCR (qPCR). Furthermore, we analyzed the expression of the cytokine IL-1β.

## Methods

### Ethics statement

The study followed the principles of the Declaration of Helsinki, was approved by the Ethics Committee of the LMU Munich, and has been registered by the German Clinical Trial Register (Registration Trial DRKS00005954).

### Subjects

Subject recruitment was estimated for 2 years. Patients presenting to our Department of Pain Medicine with chronic pain who met the inclusion criteria, as well as healthy volunteers, were enrolled after written informed consent.

### Inclusion criteria

Patients suffering from chronic non-specific low back pain (CLBP) without any signs of neuropathic pain components and patients suffering from neuropathic pain (NeP) were included. CLBP was defined as persistent low back pain not attributable to a detectable pathology (e.g. infection, tumor, osteoporosis, trauma, inflammatory disorder, or radicular syndrome). NeP was diagnosed according to its international definition (“pain caused by a lesion or disease of the somatosensory nervous system”). All patients were assessed by detailed pain history, physical examination, and the PainDETECT-questionnaire. This questionnaire consists of several items related to neuropathic pain symptoms (burning sensations, tingling or prickling sensations, shooting/lancinating, hyperalgesia, dysesthesia, allodynia, or paresthesia) with excellent sensitivity (85 %) and specificity (80 %) [17]. Healthy volunteers were evaluated for any history of pain. If no history of pain was detected in these individuals, they were included in the study.

### Exclusion criteria

Exclusion criteria were autoimmune, chronic systemic, inflammatory, neoplastic, or psychiatric diseases, drug abuse, and pregnancy. Patients on irregular medication with opioids, non-opioids, or co-analgesics were excluded. None of the patients had been treated with corticosteroids or had received immunomodulatory agents before or during the study. Any acute inflammatory process was ruled out by laboratory testing including serum concentration of C-reactive protein (CRP) and total and differential leukocyte count as well as measurement of the body temperature. Patients with mixed pain (nociceptive and neuropathic components), e.g., low back pain with radiculopathy, were excluded.

### Assessment of pain, stress, and depressive symptomatology

Patients rated their recalled average pain intensity both at rest and while moving using an 11-point numerical rating scale (NRS): 0 meaning “no pain” and 10 meaning “worst pain imaginable.” Self-perceived stress was evaluated using the German version of the Questionnaire for Actual Demands (“KAB”: “Kurzfragebogen zur aktuellen Beanspruchung”) in patients and in healthy volunteers. The KAB was designed to repeatedly quantify the individual’s level of acute or chronic stress. It is highly sensitive to short-term or situational changes during a stressful experience [18]. The rating on a scale ranging from 1 to 6 is based on normalized adjectives. Higher KAB values indicate the perception of increased stress levels. The center for epidemiologic studies depression scale (CES-D scale) which measures depressive symptomatology in the general population was used to assess depressive symptomatology in patients and in healthy volunteers. This scale contains 20 items to explore feelings or experiences during the past week. These 20 items belong to 4 main categories: depressed affect, positive affect, somatic complaints/retarded activity, and interpersonal experiences. Response options range from 0 to 3 for each item (0 = rarely or never, 1 = some or little of the time, 2 = moderately or much of the time, 3 = most or almost all of the time). Results range from 0 to 60, with higher values representing more depressive symptoms. Values >25 are considered pathological [19].

### Leukocyte count and cytokine assessment

Peripheral blood was collected from patients and healthy volunteers between 9:00 and 9:30 AM. Samples were assessed for differential leukocyte count by routine laboratory testing. For cytokine assessment, blood was centrifuged at 2000×*g* for 10 min to obtain cell-free serum. After centrifugation, supernatants were harvested and frozen at −80 °C until further use. IL-1β serum concentrations were determined using a human cytokine immunoassay (Myriad Rules-Based Medicine Inc., Austin, Texas, USA). The microbead assay is based on a Luminex technology and quantifies protein in a similar manner to standard sandwich ELISA techniques, with comparable sensitivity and range [20].

### Flow cytometric staining and analysis

**Fig. 1:**
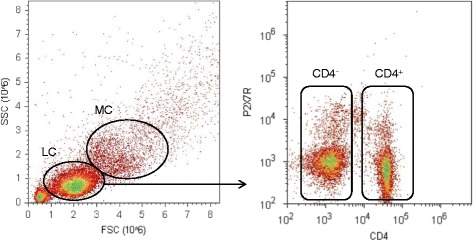
Gating strategy for the detection of lymphocytes, monocytes, CD4^+^ and CD4^−^ cells, lymphocytes (LC), and monocytes (MC) was gated according to forward scatter (FSC) and side scatter (SSC) characteristics; PerCP-labeled antihuman CD4 antibody was used to separate CD4^+^/CD4^−^ cells

**Fig. 2:**
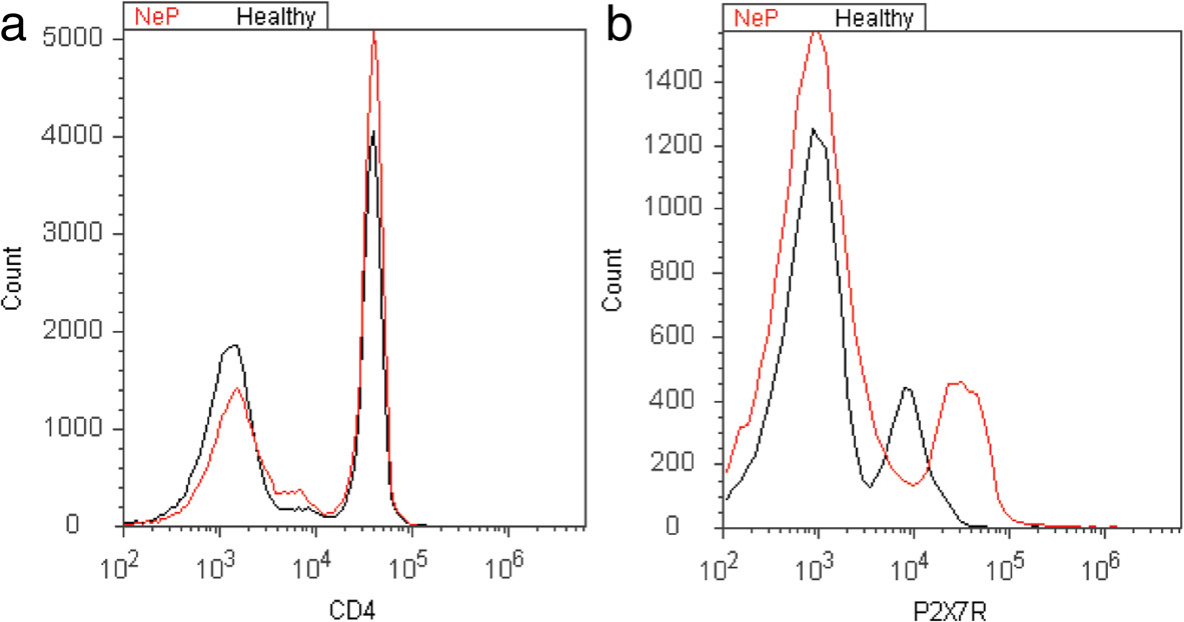
Exemplary illustration of P2X7 receptor expression. For quantitative analysis of the proportion of CD4^+^ cells (**a**) and P2X7R protein expression (**b**), cells were stained with PerCP-labeled antihuman CD4 antibody and FITC-labeled antihuman P2X7R antibody. We separately analyzed each subgroup by the mean fluorescence intensity (MFI) of P2X7R expression. Overlay histograms of representative results of P2X7R expression of one patient/healthy volunteer are displayed in histograms

Peripheral blood mononuclear cells (PBMCs) from heparinized venous blood samples were separated by Ficoll density gradient centrifugation (Sigma Aldrich, Taufkirchen, Germany). PBMCs were then cryopreserved in RPMI freezing media containing 10 % FCS and 10 % DMSO, frozen at −30 °C for 24 h, and then stored at −196 °C [21]. For FACS analyses, samples were thawed rapidly and washed twice with ice-cold FACS buffer (HBSS containing 1 % BSA and 0.1 % NaN_3_) to eliminate any remaining DMSO. For extracellular staining, cells were co-incubated with PerCP-labeled antihuman CD4 antibody (1:50, Biolegend, San Diego, CA, USA) and FITC-labeled antihuman P2X7R antibody (1:100, Alomone Labs, Jerusalem, Israel) at room temperature for 1 hour. Again, cells were washed twice with FACS buffer (400×*g*, 5 min, 4 °C) to remove excessive antibodies. P2X7R expression was then analyzed with an Attune Acoustic Focusing Cytometer (Life Technologies, Carlsbad, USA) as described by Gudipaty et al. [22]. Representative density plots and gating strategy are displayed in Fig. 1; representative histograms for evaluation of CD4^+^/CD4^−^ cells and for analyses of mean fluorescence intensity (MFI) are shown in Fig. 2.

### Quantitative RT-PCR

CD4^+^ cells were isolated from PBMCs by magnetic bead separation with the Whole Blood CD4 MicroBeads kit (MACS Miltenyi Biotec, Auburn, CA, USA) according to the manufacturer’s recommendations. Subsequently, total RNA was isolated with the mirVana miRNA Isolation Kit, followed by a DNase digest with the Turbo DNA-free kit (Ambion). Quantity and purity of the isolated RNA were measured with a NanoDrop ND-1000 spectrophotometer (Peqlab). After the amplification of total RNA using the TargetAmp 1-Round aRNA Amplification Kit (Epicentre Biotechnologies, Madison, WI, USA) and purification using an RNeasy Mini Kit (Qiagen), cDNA synthesis was performed with the SuperScript III First Strand Synthesis System (Invitrogen) and oligo-dT and random hexamer primers (Qiagen). Quantitative RT-PCR was performed in duplicates with a LightCycler 480 instrument (Roche Diagnostics, Mannheim, Germany) using LightCycler 480 Probes Master and RealTime Ready Single Assays (Roche Diagnostics). Cycling conditions were as follows: 95 °C for 10 min, 45 cycles at 95 °C for 10 s, 60 °C for 30 s, and 72 °C for 15 s. Relative mRNA expression of P2RX7 was calculated by the Relative Quantification Software (Roche Diagnostics) using an efficiency-corrected algorithm with standard curves and reference gene normalization against the reference genes succinate dehydrogenase complex subunit A (SDHA) and TATA box binding protein (TBP) as described previously [23]. Primer sequences and assay characteristics are given in Table 1.

**Table 1:** RT-PCR assay characteristics and primer sequences

Gene	Primer sequence
TBP-87	for 5′ GAACATCATGGATCAGAACAACA 3′
	rev 5′ ATAGGGATTCCGGGAGTCAT 3′
SDHA-132	for 5′ GAGGCAGGGTTTAATACAGCA 3′
	rev 5′ CCAGTTGTCCTCCTCCATGT 3′
P2RX7	Roche RealTime Ready Single Assay ID 106724

### Data analyses

Statistical analyses were performed with SigmaStat 12.0 (Systat Software, Chicago, USA). Every statistical analysis was started with testing for normal distribution using the Shapiro Wilk Test. Differences between groups were tested with the *t* test for results with normal distribution and the nonparametric Mann-Whitney Rank Sum Test for all data without normal distribution. Discrete variables were compared with the Fisher’s exact test. In order to determine significant differences between pain syndromes, we used a one-way ANOVA tests and multiple comparisons versus a control group (Holm-Sidak method). *p* values <0.05 were considered statistically significant. All results are expressed as mean ± standard deviation (SD).

## Results

### Subjects

Within 2 years of recruitment, 19 patients suffering from CLBP and 19 patients suffering from NeP who met the inclusion criteria as well as 19 pain-free volunteers were enrolled. As shown in Table 2, both groups of patients significantly differed from healthy volunteers in terms of stress level and depressive symptomatology. No significant differences were detected between the patient groups regarding pain levels at rest and during motion, as well as duration of pain (Table 2). Furthermore, statistical analysis of the number of patients receiving analgesic and coanalgesic medication revealed no significant difference between the two groups (Table 3).

**Table 2:** Patients characteristics

	Healthy	CLBP	NeP
Numbers	19	19	19
Age (years)	40 ± 11	47 ± 13	58 ± 13*
Female (%)	58 %	79 %	68 %
BMI (kg/m^2^)	23.6 ± 2.9	23.9 ± 3.1	24.6 ± 3.8
Duration of pain (years)	0.0 ± 0.0	5.9 ± 4.2*	4.5 ± 2.8*
NRS (rest)	0.0 ± 0.0	3.5 ± 2.2*	5.1 ± 2.2*
NRS (motion)	0.0 ± 0.0	4.4 ± 2.1*	6.9 ± 2.1*
KAB	1.7 ± 0.7	3.4 ± 0.9*	3.3 ± 0.9*
CES-D	2.4 ± 2.2	21.8 ± 7.3*	21.0 ± 9.6*

Patients with either CLBP or NeP significantly differed from healthy volunteers in terms of duration of pain, NRS, KAB, and CES-D. However, no significant differences were detected between CLBP and NeP patients for any of the parameters. Data are presented as mean ± SD, *n* = 19, *p < 0.05 versus healthy in paired Student’s *t* test (NeP) and Mann-Whitney Rank Sum Test (CLBP)

*BMI* body mass index; *NRS* (rest/motion) numeric rating scale (0–10) of pain, 0: “no pain,” 10: “worst pain imaginable,” *KAB* questionnaire for self-perceived stress ranging from 1 (no stress) to 6 (max. stress), *CES-D* center for epidemiologic studies depression scale, *CLBP* chronic low back pain, *NeP* neuropathic pain (symmetrical polyneuropathy, peripheral mononeuropathy, postherpetic neuralgia, orofacial pain)

**Table 3:** Patients’ medication at the beginning of the study

Medication at beginning of the study	CLBP (*n* = 19)	NeP (*n* = 19)
Ibuprofen (no. (%))	3 (16)	2 (11)
Diclofenac (no. (%))	2 (11)	3 (16)
Paracetamol (no. (%))	3 (16)	2 (11)
Metamizole (no. (%))	1 (5)	1 (5)
Opioids (no. (%))	2 (11)	3 (16)
Pregabalin (no. (%))	0 (0)	1 (5)
Duloxetine (no. (%))	0 (0)	1 (5)

There is no significant difference in the intake of analgesics or coanalgesics in patients with CLBP and NeP. Data are presented as mean ± SD, *n* = 19, statistical testing was performed using Fisher’s exact test

*CLBP* chronic low back pain; *NeP* neuropathic pain (symmetrical polyneuropathy, peripheral mononeuropathy, postherpetic neuralgia, orofacial pain)

### Differential blood count and quantification of CD4^+^ cells

**Fig. 3:**
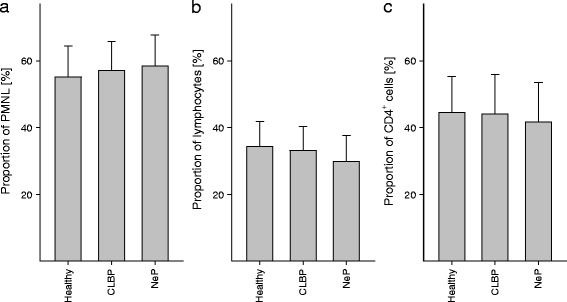
Differential blood count and quantification of CD4^+^ cells. In order to avoid misinterpretation of potentially elevated P2X7R protein expression based on different cell counts, we quantified numbers of polymorphonuclear leukocytes (**a**), lymphocytes (**b**), and CD4^+^ cells (**c**). No differences were found between patients suffering from CLBP, NeP, and healthy volunteers. Data are presented as mean ± SD, *n* = 19, *p < 0.05 in one-way ANOVA with multiple comparisons versus healthy volunteers (Holm-Sidak method)

We quantified the number of neutrophil granulocytes, representing an essential part of the innate immune system, as well as total lymphocytes and CD4^+^ T cells as key players of the adaptive immune response. As shown in Fig. 3, numbers of polymorphonuclear leukocytes (CLBP: 57.1 ± 8.7 %, NeP: 58.4 ± 9.1 %, healthy volunteers: 55.2 ± 9.0; n.s.), total lymphocytes (CLBP: 33.2 ± 6.9 %, NeP: 29.9 ± 7.6 %, healthy volunteers: 34.4 ± 7.2; n.s.), and CD4^+^ T cells (CLBP: 44.1 ± 11.4 %, NeP: 41.7 ± 11.3 %, healthy volunteers: 44.5 ± 10.5; n.s.) did not differ between patients suffering from CLBP, NeP, or healthy volunteers.

### P2RX7 mRNA expression is increased in patients with NeP, but not in CLBP

**Fig. 4:**
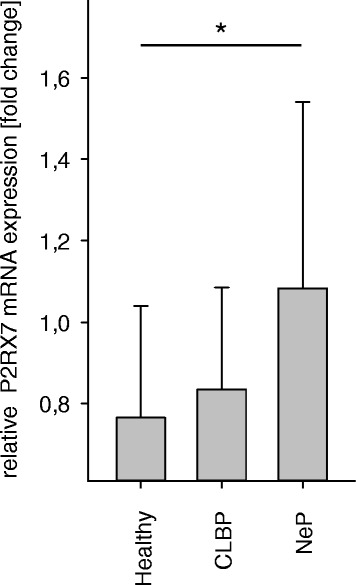
P2RX7 mRNA expression is exclusively increased in patients with neuropathic pain. To confirm the flow cytometric observations with elevated P2X7R protein expression, predominantly on CD4^+^ cells and only in NeP, we determined the relative P2RX7 mRNA expression of CD4^+^ cells by qPCR. Affirmatively, increased mRNA levels were consistent with the flow cytometric analyses and exclusively elevated in patients with NeP. (*p* = 0.038). *p < 0.05. Data are presented as mean ± SD, *n* = 19, *p < 0.05 in one-way ANOVA with multiple comparisons versus healthy volunteers (Holm-Sidak method)

The relative expression of P2RX7 mRNA was determined by qPCR. Compared to healthy volunteers, significantly elevated mRNA levels (1.6-fold) were detected in patients with NeP (NeP: 1.6 ± 0.6, healthy volunteers 1.0 ± 0.3, *p* < 0.05; Fig. 4), while only a mild increase of P2RX7 mRNA expression (1.1-fold) was found in patients with CLBP (CLBP: 1.1 ± 0.6, healthy volunteers 1.0 ± 0.3, n.s.; Fig. 4).

### P2X7R protein expression is significantly increased on lymphocytes and monocytes of patients with NeP

**Fig. 5:**
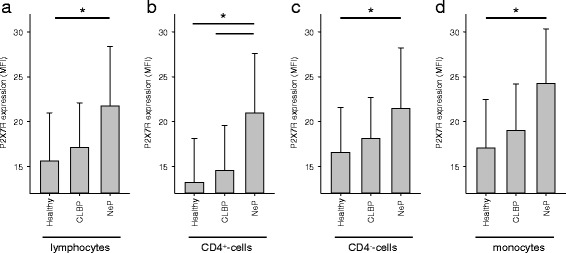
P2X7R expression on lymphocytes (**a**), CD4^+^ (**b**) and CD4^−^ cells (**c**), and monocytes (**d**) is elevated in patients with neuropathic pain. PBMCs were stained with PerCP-labeled antihuman CD4 antibody and FITC-labeled antihuman P2X7R antibody and analyzed by FACS. The results show a significantly elevated P2X7R expression only in patients suffering from neuropathic pain on the analyzed cell type (lymphocytes (*p* = 0.009), CD4^+^ cells (*p* < 0.001), CD4^−^ cells (*p* = 0.039), and monocytes (*p* = 0.002)). Data are presented as mean ± SD, *n* = 19, *p < 0.05 in one-way ANOVA with multiple comparisons versus healthy volunteers (Holm-Sidak method)

Consistent with the results on mRNA expression, FACS results (MFI) revealed that compared to healthy volunteers, P2X7R protein expression levels on lymphocytes (NeP: 21.8 ± 6.5, healthy volunteers: 15.6 ± 5.2; *p* = 0.009; Fig. 5a) and monocytes (NeP: 24.6 ± 6.2, healthy volunteers: 17.0 ± 5.4; *p* = 0.002; Fig. 5d) were significantly enhanced in patients with NeP, but not in patients with CLBP. This P2X7R upregulation was detected on both CD4^+^ monocyte (NeP: 21.0 ± 6.4, healthy volunteers: 13.2 ± 4.8; *p* < 0.001; Fig. 5b) and CD4^−^ monocyte (NeP: 21.5 ± 6.5, healthy volunteers: 16.6 ± 4.9; *p* = 0.039; Fig. 5d) cells.

### Differential IL-1β levels in patients with neuropathic pain and CLBP

**Fig. 6:**
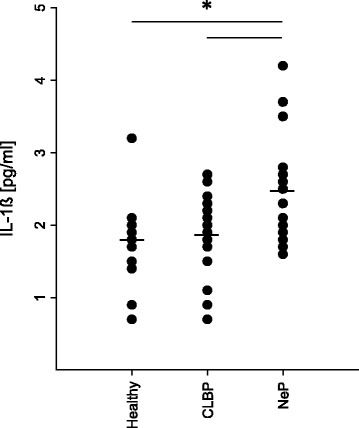
IL-1β levels are elevated in patients with neuropathic pain. As IL-1β was shown to be the key mediator in P2X7R-pain interplay, we analyzed serum levels of this pro-inflammatory and pro-nociceptive cytokine by multiplex immune assay. Affirmatively to the results with elevated P2X7Rs, we solely found significant increased serum levels in the peripheral blood of patients with NeP *p < 0.05. Data are presented as mean ± SD, *n* = 19, *p < 0.05 in one-way ANOVA with multiple comparisons versus healthy volunteers (Holm-Sidak method)

In a number of preclinical studies, IL-1β has been shown to be mainly involved in neurodegeneration, inflammation, and pain [1, 10] and to be a key mediator of the P2X7R-pain interplay. In order to find out whether the observed differences in P2X7R expression between patients with CLBP and NeP are also reflected by IL-1β, we analyzed serum levels of this pro-inflammatory and pro-nociceptive cytokine by multiplex enzyme-linked immunoassay. Concomitant with the P2X7R elevation, we found significantly increased serum levels of IL-1β (1.4-fold) only in the peripheral blood of patients suffering from neuropathic pain (Fig. 6).

### Confounding analyses

Since P2X7R expression has been associated with various diseases, we tested a set of potentially confounding variables for significant differences in distribution between healthy volunteers and patients [24, 25]. As elevated receptor expression is associated with depression and anxiety, we tested the CES-D depression scores and KAB values. Healthy volunteers showed significantly lower depression and stress scores, but no differences were found between patients with nociceptive and neuropathic pain. Age differed significantly between patients and healthy volunteers. However, a potential confounding effect was excluded by investigating the correlation between age and P2X7R levels (no significant correlation for both, Pearson’s and Spearman’s correlation test; *r* = 0.18, *p* = 0.16 and rho = 0.17, *p* = 0.18, respectively), as well as checking P2X7R levels in age-matched subgroups that yielded similar results as the main analysis. Moreover, no correlation was found regarding gender and receptor expression. Subgroup analysis of neuropathic pain syndromes revealed no differences in P2X7R expression between peripheral polyneuropathy/mononeuropathy, postherpetic neuralgia or orofacial pain.

## Discussion

In this study, we found a significantly increased expression of P2X7R mRNA and protein in lymphocytes and monocytes as well as higher IL-1β serum levels in patients suffering from NeP, but not in those with nociceptive CLBP. These results might point to an important role of P2X7R and IL-1β in the pathogenesis and maintenance of NeP.

Chronic pain is a global health problem, affecting up to 60 % of the population [26]. Over the last years, significant effort has been made to investigate endogenous pain-modulating factors [27]. In various animal models of nociceptive, inflammatory, and neuropathic pain, the endogenous receptor P2X7 was the focus of interest [2–5]. Recent data indicate that nociceptive information from the periphery to the CNS is transmitted through various ion channels and receptor pathways [28, 29]. The ATP-sensitive P2X7R, which is particularly localized on immune and microglial cells, is part of this reporter system [29]. In response to inflammation or cellular damage, ATP activates P2X7R, which represents an important step in the transmission of sensory information to the central nervous system [4, 30]. Recent studies suggest that P2X7R is involved in the pathogenesis of neurological disorders such as epilepsy, stroke, neuralgia, multiple sclerosis, Alzheimer’s disease, Parkinson’s disease, and Huntington’s disease [31]. Moreover, the P2X7R is associated with mood disorders like major depression or anxiety [31, 32]. Upon activation, P2X7R triggers a series of physiological events that culminate in the posttranscriptional processing and release of IL-1β from monocytes [10]. IL-1β is a pro-inflammatory and pro-nociceptive cytokine which was shown to be a key mediator in chronic pain [33]. In addition, there is increasing evidence that enhanced release of IL-1β after P2X7R activation antagonizes morphine analgesia and accounts for the development of morphine tolerance, which may partly explain the insufficient effect of opioids in a considerable number of NeP patients [34]. IL-1β induces the transcription of cyclo-oxygenase 2 (COX-2) and nitric oxide synthase (iNOS), which play a central role in the generation and maintenance of pain [35, 36]. Within clinical settings, the efficacy of agents to treat neuropathic pain is variable. While COX-2 inhibitors are particularly effective against the inflammatory component of neuropathic pain, their effect on the intensity of pain is generally not satisfactory [37]. Contrary, antiepileptic drugs and antidepressants are useful to modulate the intensity of pain, but rather inefficient to treat the inflammatory component [38]. P2X7R and IL-1β are known to modulate inflammation and nociception, which recently led to the discovery of pharmacological agents selectively blocking P2X7R [30]. Genetic modulation or pharmacological blockade of P2X7R induces a regression of symptoms in animal models of neurological disorders and reduces the intensity of inflammatory and neuropathic pain in mice [4, 39, 40].

These findings are consistent with our results demonstrating an increased expression of lymphocyte and monocyte P2X7R and IL-1β in patients suffering from NeP. It is not surprising, that only slight elevations of P2X7R protein, P2RX7 mRNA expression and of IL-1β levels were found in patients with CLBP, as CLBP is usually not associated with significant immune activation. These findings might point to a minor role of the P2X7R/IL-1β interplay in the pathophysiology of CLBP. This assumption is supported by recent research, showing a communication link between the immune system and the CNS [1, 15]. Lesion of a peripheral nerve leads to both a transition of microglia to the side of damage and an infiltration of immune cells in the vicinity of the synapse between primary afferent fibers and nociceptive neurons in the dorsal horn of the spinal cord [41, 42]. These activated immune cells release many pro-inflammatory mediators, such as IL-1β which cross the blood-brain barrier [15] and modulate pain intensity [40, 43]. A crucial regulator of IL-1β release is P2X7R [10]. Peripheral knock-down of P2X7R in mice leads to a significant decrease of IL-1β release and reduction of pain intensity [40].

Since elevated P2X7R expression has also been associated with mood disorders such as depression and anxiety, we tested the CES-D depression scores and KAB values as potentially confounding variables. Healthy volunteers had significantly lower depression and stress scores than both patient subgroups, but no differences were found between patients suffering from CLBP or NeP. In order to exclude further factors being responsible for the different expression of P2X7R/IL-1β in pain syndromes, we performed confounder-analyses. Although age differed between patients and healthy volunteers, a potential confounding effect could be excluded. Furthermore, we found that gender did not correlate with the expression of P2X7R or IL-1β either. Regarding subgroups of NeP, no differences with respect to the P2X7R expression were found between patients suffering from peripheral polyneuropathy/mononeuropathy, postherpetic neuralgia or orofacial pain. One limitation of our study is that the analysis of P2X7R protein and mRNA expression was performed on lymphocytes, whereas IL-1β levels were determined in the peripheral blood. Since P2X7R is also expressed on the surface of other immune cells such as macrophages, which are also a major source of IL-1β production, further studies are needed to clearly define the source of elevated IL1β levels. Furthermore, it would be interesting to take the monocytes followed by LPS priming and ATP challenge to demonstrate different IL-1β release between groups.

## Conclusions

In conclusion, we here report that, in patients with NeP, P2X7R expression is significantly elevated. Activation of P2X7 has been shown to result in IL-1β release. Thus, based on our data, we propose the hypothesis that increased P2X7R expression leads to increased IL-1β blood levels, which may either predispose or maintain neuropathic pain. This P2X7R-driven inflammatory component seems to be absent in patients with CLBP. Our results suggest a major role of the purinergic-receptor/cytokine-interplay in NeP and may help identify patients who might benefit from P2X7R modulating treatment approaches.

## Abbreviations

BMI: body mass index
CES-D scale: center for epidemiologic studies depression scale
CLBP: chronic low back pain
CNS: central nervous system
COX-2: cyclo-oxygenase 2
CRP: C-reactive protein
FACS: fluorescent-activated cell sorting
FSC: forward scatter
IL: interleukine
iNOS: nitric oxide synthase
KAB: Kurzfragebogen zur aktuellen Beanspruchung
LC: lymphocytes
MC: monocytes
MFI: mean fluorescence intensity
NeP: neuropathic pain
NRS: numerical rating scale
P2XR: purinergic P2X receptor
PBMC: peripheral blood mononuclear cell
RT-PCR: quantitative real-time PCR (qPCR)
SD: standard deviation
SDHA: succinate dehydrogenase complex subunit A
SSC: side scatter
TBP: TATA box binding protein
Treg: regulatory T cell
